# Balloon dilatation for congenital esophageal stenosis associated with esophageal atresia

**DOI:** 10.1007/s00383-024-05652-w

**Published:** 2024-03-22

**Authors:** Koichi Deguchi, Masafumi Kamiyama, Kazunori Masahata, Motonari Nomura, Miho Watanabe, Takehisa Ueno, Yuko Tazuke, Hiroomi Okuyama

**Affiliations:** Department of Pediatric Surgery, Graduate School of Medicine, Osaka University, 2-2, Yamadaoka, Suita-shi, Osaka Japan

**Keywords:** Congenital esophageal stenosis, Esophageal atresia and tracheoesophageal fistula, Balloon dilatation, Surgical resection, Anastomotic stricture

## Abstract

**Purpose:**

Congenital esophageal stenosis (CES) associated with esophageal atresia (EA) is rare, and no standard treatment has been established. We reviewed cases of EA-associated CES to assess the clinical characteristics and treatment outcomes, especially the feasibility of endoscopic dilatation.

**Methods:**

We retrospectively examined patients with EA-associated CES. We also compared treatment outcomes of EA-associated CES with those of EA patients without CES who developed postoperative anastomotic stricture.

**Results:**

Among 44 patients with EA, ten had CES (23%). Postoperative complications were not significantly different between EA patients with CES and those without CES but with anastomotic stricture. All CES patients underwent balloon dilatation as initial treatment. Eight of nine patients (89%) were successfully treated by dilatation only, and one patient underwent surgical resection. The median number of balloon dilatations for CES was five (2–17), which was higher than that for anastomotic stricture in patients without CES (*p* = 0.012). Esophageal perforation occurred in five patients with CES (5/9, 56%) after dilatation, but all perforations were successfully managed conservatively with an uneventful post-dilatation course.

**Conclusions:**

Twenty-three percent of patients with EA had CES. Although balloon dilatation for EA-associated CES required multiple treatments and carried a risk of perforation, balloon dilatation showed an 89% success rate and all perforations could be managed conservatively.

## Introduction

Congenital esophageal stenosis (CES) is a rare malformation that results from intramural narrowing of the lower esophagus [1]. Three types of CES have been described in the literature: tracheobronchial remnants (TBR), fibromuscular hypertrophy (FMH), and membranous webs (MW) [2]. Recently, increasing numbers of studies have recognized the association between CES and esophageal atresia (EA) [3, 4].

There is currently no general agreement on the optimal management of CES associated with EA (EA-associated CES) [5, 6]. In most cases, surgical resection was the treatment of choice for CES lesions because dilation was unsuccessful [5]. However, in EA-associated CES, the resection of CES lesions is sometimes complicated due to reduced blood supply in the lower esophagus or preexisting intrathoracic adhesions caused by thoracotomy for EA repair. Therefore, safer options such as balloon dilatation may benefit eligible patients.

The primary aim of this retrospective study was to investigate the efficacy of balloon dilatation for EA-associated CES at our center. The secondary aim was to elucidate the characteristics of patients with EA-associated CES by comparing to EA patients without CES who developed postoperative anastomotic stricture.

## Methods

### Patients

We retrospectively reviewed 44 patients who underwent surgery for EA at our institution between January 2000 and December 2021. Patient characteristics, specifically sex, gestational age, birth weight, type of EA, associated anomalies, clinical symptoms, diagnostic modalities, age at diagnosis, initial management, and management outcomes were recorded. EA-associated CES was diagnosed when persistent radiographic evidence of esophageal narrowing in a different location than the anastomosis was observed since the neonatal period, or when histologic evidence of CES was found in the stenotic segment. The disease duration was defined by the days from the first symptom to the last treatment session. Postoperative complications were categorized according to the Clavien–Dindo classification [7], and complications of grade III or above were recorded. CES was evaluated by radiologic esophagography, and detailed information, including location, morphology (tapered or abrupt narrowing), and the diameter of the stenotic site, was recorded. The exclusion criteria were deficient clinical information, such as inaccessible imaging data, and conditions initially treated as CES, but later identified as other pathologies such as achalasia, tumor, or acquired stenosis. To further clarify the characteristics of individuals with EA-associated CES, we compared two cohorts: EA patients with CES, and EA patients without CES who developed anastomotic stricture after EA repair.

### Balloon dilatation technique

Esophagogastroscopy was first performed under general anesthesia to examine the stenotic site. The esophageal balloon was then introduced using the through-the-scope or over-the-wire technique, and dilatation was performed fluoroscopically. The inflation time was 1–3 min with both techniques. Additional dilatation sessions were performed using a larger-diameter balloon when the dilatation was insufficient. Dilatation was terminated when the balloon notch at the stenotic site disappeared, or if esophageal injury of the mucosa or muscular layer was confirmed by esophagogastroscopy. The diagnosis of esophageal perforation was made if chest X-ray or computed tomographic imaging revealed pneumomediastinum or if contrast leakage was observed by esophagography. In patients without radiologic evidence, suspected esophageal perforation was defined based on fever over 38 °C and increased C-reactive protein levels without signs of aspiration pneumonia.

### Statistics and ethical considerations

All statistical analyses were performed using GraphPad Prism (version 9.00; GraphPad Software, San Diego, CA, USA). Continuous variables are expressed as median and interquartile range unless otherwise specified. Statistical significance was calculated using the chi-square test and Mann–Whitney *U* test. A value of *p* < 0.05 was considered to be statistically significant. This study was approved by the institutional review board of our hospital (IRB 20567).

## Results

### Patient characteristics

The characteristics of patients with EA-associated CES (EA with CES group) and with EA and anastomotic stricture (AS group) are summarized and compared in Table 1. Overall, the incidence of CES in patients with EA was 10 of 44 (23%); these 10 patients were included in the EA with CES group, and the 19 patients without CES but who developed anastomotic stricture postoperatively and required intervention to establish oral feeding were assigned to the AS group. 6 of 10 (60%) patients in the EA with CES group had long-gap EA, compared to 7 of 19 (37%) patients in the AS group, a difference that was not statistically significant (*p* = 0.27). Regarding the operative approach, esophageal elongation was performed in 5 patients (2 in the EA with CES group and 3 in the AS group).

**Table 1:** Comparison of patient characteristics between esophageal atresia with or without associated congenital esophageal stenosis

	EA with CES	EA with anastomotic stricture^b^	*p* value
Number	10	19	
Sex (male:female)	4:6	7:12	0.99
Gestational age (weeks)	37.1 (36.0–39.0)	38.3 (37.3–39.8)	0.092
Birth weight (g)	2187 (1724–2564)	2266 (2064–2494)	0.51
Premature	3 (30%)	4 (21%)	0.66
APGAR	8 (4–8)/9 (6.25–9)	8 (5–8)/9 (8–9)	0.84/0.79
Type of EA			
Type A	2 (20%)	3 (16%)	0.99
Type C	8 (80%)	16 (84%)	
Long gap^a^	6 (60%)	7 (37%)	0.27
Associated malformations			
All	4 (40%)	16 (80%)	0.051
Cardiac/major	1	6	
Cardiac/minor	3	0	
Chromosomal	0	1	
Duodenal	0	1	
Anorectal	4	1	
VACTERL association	2	2	
Cryptorchism	2	0	
Trachea	2	4	
Prenatal diagnosis	6 (60%)	6 (32%)	0.26
Esophageal elongation	2 (20%)	3 (16%)	0.99
Gastrostomy	6 (60%)	12 (67%)	0.99
Operative time (min)	211 (187.5–337.8)	219 (177–288)	0.99
Bleeding (g)	5.0 (0.1–22.5)	12.5 (5.0–33.8)	0.26
Follow-up period (months)	85.8 (39.2–199.9)	205.8 (140.6–290.9)	0.014

Values are presented as number only, median (interquartile range), or number (%)

*EA* esophageal atresia, *CES* congenital esophageal stenosis

^a^Esophageal gap greater than three vertebral bodies

^b^Patients with postoperative anastomotic stricture who required intervention to establish oral feeding

### Clinical presentations

Table 2 summarizes the clinical presentations of the EA with CES group (*n* = 10) in comparison to the AS group (*n* = 19). Postoperative contrast esophagograms were used to diagnose all cases of EA-associated CES. The mean ages at initial symptom presentation and initial diagnosis were lower in the EA with CES group than in the AS group (89, 110 days vs. 203, 224 days, respectively), although the difference was not statistically significant. Seven of ten patients in the EA with CES group were diagnosed before 6 months of age, and there was only one case of delayed diagnosis (at 610 days of age). The typical clinical symptoms in both groups included vomiting and difficulty swallowing solid or liquid food. One patient in the EA with CES group and 2 in the AS group suffered from recurrent aspiration.

**Table 2:** Comparison of presentation between esophageal atresia with associated congenital esophageal stenosis or anastomotic stricture

	EA with CES (*n* = 10)	EA with anastomotic stricture (*n* = 19)	*p* value
Age at first symptom (days)	89 (45–270)	203 (27–512)	0.38
Age at diagnosis (days)	110 (52–291)	224 (32–535)	0.42
Symptom			
Vomiting	6	7	
Difficulty in swallowing solid food	6	8	
Difficulty in swallowing liquid food	3	10	
Foreign body impaction	1	1	
Recurrent aspiration	1	2	
Location			
Upper esophagus	0	3	0.0002
Middle esophagus	2	15	
Lower esophagus	8	1	
Morphology of esophageal narrowing			
Tapered	8	12	0.43
Abrupt	2	7	

Values are presented as number only, median (interquartile range), or number (%)

*EA* esophageal atresia, *CES* congenital esophageal stenosis

Regarding esophageal narrowing, all cases of EA-associated CES occurred distal to the anastomotic site, and the majority were located in the lower esophagus (*p* = 0.0002). Tapered narrowing was more common than abrupt narrowing in both groups (*p* = 0.43).

### Management and outcomes

Fluoroscopy- or endoscopy-guided balloon dilatation was attempted as the initial treatment in all patients. The management and outcomes of patients with EA-associated CES or AS are summarized and compared in Table 3. 9 of 10 patients in the EA with CES group and 18 of 19 patients in the AS group underwent balloon dilatation. Two patients (one in the EA with CES group and the other in the AS group) underwent no treatment because of coexisting severe cardiac malformations and oral feeding intolerance, and both required parenteral nutrition. The median disease duration in the EA with CES group was 500 days, which was significantly longer than in the AS group (*p* = 0.0018). Although a larger balloon (median 18 mm) was required in the EA with CES group than in the AS group (*p* = 0.043), the median number of repeat dilatations in both groups was not significantly different (5 vs. 3, *p* = 0.17).

**Table 3:** Comparison of management and outcomes between esophageal atresia with associated congenital esophageal stenosis or anastomotic stricture

	EA with CES (*n* = 10)	EA with anastomotic stricture (*n* = 19)	*p* value
Age at first dilatation (days)	117 (29–298)	231 (39–542)	0.42
Patients without treatment			
Asymptomatic	0	0	
No oral food intake	1	1	
Disease duration (days)	500 (366–1893)	49 (14–161)	0.0018
Balloon size (mm)	18 (14–20)	13 (8–17)	0.043
Steroid use			
Local injection	4 (44%)	7 (39%)	0.99
Systemic	0	0	
Number of dilatations	5 (2–9)	3 (1–4)	0.17
Overall success rate of dilatation	89% (8/9)	94% (17/18)	0.99
Complication of dilatation			
Perforation	5 (56%)	1 (6%)	0.0080
Aspiration	0	4 (21%)	0.13
Management after perforation			
Medical	5	1	
Surgical	0	0	
Resection	1 (11%)	1 (6%)	0.99

Values are presented as number only, median (interquartile range), or number (%)

*EA* esophageal atresia, *CES* congenital esophageal stenosis

Overall, 89% of patients in the EA with CES group and 94% in the AS group were successfully managed by balloon dilatation only, and oral intake was established (Table 3). Complications associated with balloon dilatation in both groups included esophageal perforation and aspiration pneumonia. Esophageal perforation (including suspected cases) due to balloon dilatation occurred in 6 patients, and the incidence was significantly higher (5/9 patients, 56%) in the EA with CES group than in the AS group (1/19 patients, 6%, *p* = 0.008). All perforations following balloon dilatation were associated with minor symptoms such as increased body temperature, leukocytosis, and elevated C-reactive protein levels for several days, and they could be managed nonoperatively with uneventful recovery. Interestingly, stenotic symptoms resolved after esophageal perforation in two of five EA-associated CES patients. Postoperative aspiration pneumonia predominantly occurred in the AS group (4/19 patients, 22%) in our cohort (*p* = 0.13).

Two patients (one in the EA with CES group and the other in the AS group) with recurring narrowing that was refractory to repeated balloon dilatations underwent surgical resection. In the patient with EA-associated CES, we performed surgical resection of the stenotic lesion with the Collis–Nissen procedure [8, 9]. Histologic examination revealed TBR. Surgical resection resulted in good oral intake for the patient in the AS group, but the patient with EA-associated CES developed esophageal narrowing at the fundoplication site postoperatively. Eventually, this patient also became capable of regular oral intake after occasional balloon dilatations.

### Postoperative complications of EA repair according to the presence of CES

Finally, we compared the details of postoperative complications after EA repair to elucidate the effect of CES presence on postoperative outcome (Table 4). Overall, the groups showed no significant differences in the incidence of postoperative complications, including anastomotic leakage, stricture, recurrent TEF, reoperation, gastroesophageal reflux, and recurrent aspiration, although anastomotic leakage was more common in the AS group (9/19 patients, 47%, *p* = 0.098).

**Table 4:** Comparison of complications after repair of esophageal atresia with associated congenital esophageal stenosis or anastomotic stricture

	EA with CES (*n* = 10)	EA with anastomotic stricture (*n* = 19)	*p* value
Anastomotic leakage			
All	1 (10%)	9 (47%)	0.098
Major	0	3 (16%)	0.53
Recurrent TEF	0	0	0.99
Reoperation for EA	1	3	0.99
GER^a^			
Surgically managed	3 (30%)	5 (26%)	0.99
Recurrent aspiration	3 (30%)	2 (11%)	0.40

Values are presented as number only, median (interquartile range), or number (%)

*EA* esophageal atresia, *CES* congenital esophageal stenosis

^a^Patients with GER who required surgical intervention

## Discussion

Although the association of CES with EA had been considered rare [6, 10], recent publications and a systematic review suggested that the association has been underrecognized [3–5]. Surgical resection has been the predominant approach for treating EA-associated CES [5], and no studies have explored the efficacy of balloon dilatation. Here we reviewed EA-associated CES patients treated at our institution. We found that 10 of 44 EA patients (23%) had associated CES. Balloon dilatation for EA-associated CES was safe and effective in all but one (11%) patient with severe CES who required surgical resection due to TBR, although we experienced several cases of esophageal perforation following balloon dilatation. We found no unique epidemiologic characteristics in patients with EA-associated CES. The incidence of postoperative complications in these patients was similar to those without CES.

The management of CES is primarily divided into surgical and non-surgical, including balloon dilatation, and the pathologic type of CES is considered when choosing treatment strategies [11]. Balloon dilatation has been considered to be effective for patients with MW and some with FMH, but not for those with TBR [12, 13]. A more recent study suggested that dilatation may be applicable irrespective of pathologic type [14]. In the setting of EA-associated CES, surgical resection has been preferred in previous research. Two studies reported that dilatation was effective in only 22% and 11% of cases, respectively, and was associated with a considerable risk of perforation [4, 15]. These studies speculated that the low efficacy and high perforation rate of dilatation may be due to an association between TBR-type CES and EA. Another study advocating surgical resection showed that surgery for CES after EA repair was associated with an increased incidence of postoperative complications [16, 17]. By contrast, Newman et al. and McCann et al. treated 89% and 65% of patients with dilatation only [6, 18]. They found that dilatation was associated with a higher incidence of perforation (18–22%), although most perforated patients underwent bougie dilatation rather than balloon dilatation [6, 18].

In our analysis, dilatation for EA-associated CES was effective in 89% of cases, and only one patient with TBR stenosis required surgical resection. In agreement with McCann et al. and other case serieses [4, 15, 18], our CES patients also experienced a high incidence (56%) of esophageal perforation after balloon dilatation, and this incidence was higher than that in EA patients with AS. However, all perforated patients could be managed conservatively with an uneventful postoperative course, indicating that more patients with EA and associated CES can be managed less invasively by dilatation than previously considered.

Our high success rate in managing EA-associated CES with balloon dilatation contradicts the report by Kawahara et al., which suggested a high association between TBR stenosis (3/11 patients, 27%) and a low efficacy of dilatation in EA-associated CES [15]. Although a direct comparison is not possible because balloon dilatation cannot obtain specimens for pathologic diagnosis, our patients with EA-associated CES were more likely to have tapered than abrupt narrowing (8/10 patients, 80%, Table 2), indicating less significant stenosis in our cohort [19]. These results are generally in line with our previous multi-center study: patients with EA-associated CES had less severe narrowing than those who had CES not associated with EA (isolated CES), as evaluated by the angles formed by the esophageal wall during contrast esophagography [20]. Although we only encountered a single patient with TBR requiring surgical resection, resection of the stenotic segment should be considered when multiple attempts at dilatation prove refractory and TBR is suspected based on an imaging modality, such as endoscopic ultrasound. Future studies should clarify the distribution of the type of stenosis in EA-associated CES, which will determine the optimal treatment strategy for this patient group.

To assess the characteristics of EA-associated CES or the influence of CES on the clinical course of EA patients, we analyzed background characteristics and outcomes of patients with EA-associated CES. Data of these patients were compared with those of EA patients with AS. As also reported by McCann et al. [18], no specific background characteristics of EA-associated CES were identified, and no attributes predicted the presence of CES following EA repair. However, we found slightly more long-gap cases and fewer associated malformations in the EA-associated CES patients (Table 1). Therefore, clinicians should be aware of the potential association of CES with EA. The EA with CES group had fewer cardiac and more anorectal anomalies (Table 1). Some previous studies suggested that EA-associated CES might result in increased postoperative complications at the anastomotic site [10, 15, 21], although a more recent study, like our own, experienced no such cases [4]. The disease duration in patients with EA-associated CES, defined as days from the first symptom to the last treatment session, was significantly longer than in patients with EA and AS, suggesting that CES was more refractory to dilatation than AS (Table 3).

## Conclusions

In conclusion, CES occurred in 23% of our EA cohort. Balloon dilatation for EA-associated CES achieved a good outcome with an overall success rate of 89%, and complications associated with dilatation were minimal. Although most of our cohort did not have severe EA-associated CES, balloon dilatation may be a safe first-line treatment option in patients with EA-associated CES, given the limitation of current medical technology to recognize the histological type of CES preoperatively.

## Data Availability

The data that support the findings of this study are available from the corresponding author, upon reasonable request.
